# Cost-effectiveness analysis of flucytosine as induction therapy in the treatment of cryptococcal meningitis in HIV-infected adults in South Africa

**DOI:** 10.1186/s12913-021-06268-9

**Published:** 2021-04-06

**Authors:** Jacqui Miot, Trudy Leong, Simbarashe Takuva, Andrew Parrish, Halima Dawood

**Affiliations:** 1 Health Economics and Epidemiology Research Office, School of Clinical Medicine, Faculty of Health Sciences, University of the Witwatersrand, Johannesburg, South Africa; 2 National Department of Health, Affordable Medicines Directorate, Essential Drugs Programme, Pretoria, South Africa; 3 Perinatal HIV Research Unit, Faculty of Health Sciences, University of the Witwatersrand, Johannesburg, South Africa; 4 School of Health Systems and Public Health, Faculty of Health Sciences, University of Pretoria, Pretoria, South Africa; 5 Department of Internal Medicine, Cecilia Makiwane Hospital, East London, South Africa; 6 Department of Medicine, Greys Hospital, Pietermaritzburg, KwaZulu-Natal South Africa; 7 Caprisa, University of KwaZulu-Natal, Durban, South Africa

**Keywords:** Cost-effective, Cost impact, 5-flucytosine, Cryptococcal meningitis, HIV

## Abstract

**Background:**

Cryptococcal meningitis in HIV-infected patients in sub-Saharan Africa accounts for three-quarters of the global cases and 135,000 deaths per annum. Current treatment includes the use of fluconazole and amphotericin B. Recent evidence has shown that the synergistic use of flucytosine improves efficacy and reduces toxicity, however affordability and availability has hampered access to flucytosine in many countries. This study investigated the evidence and cost implications of introducing flucytosine as induction therapy for cryptococcal meningitis in HIV-infected adults in South Africa.

**Methods:**

A decision analytic cost-effectiveness and cost impact model was developed based on survival estimates from the ACTA trial and local costs for flucytosine as induction therapy in HIV-infected adults with cryptococcal meningitis in a public sector setting in South Africa. The model considered five treatment arms: (a) standard of care; 2-week course amphotericin B/fluconazole (2wk AmBd/Flu), (b) 2-week course amphotericin B/flucytosine (2wk AmBd/5FC), (c) short course; 1-week course amphotericin B/flucytosine (1wk AmBd/5FC) (d) oral course; 2-week oral fluconazole/flucytosine (oral) and e) 1-week course amphotericin B/fluconazole (1wk AmBd/Flu). A sensitivity analysis was conducted on key variables.

**Results:**

The highest total treatment costs are in the 2-week AmBd/5FC arm followed by the 2-week oral regimen, the 1-week AmBd/5FC, then standard of care with the lowest cost in the 1-week AmBd/Flu arm. Compared to the lowest cost option the 1-week flucytosine course is most cost-effective at USD119/QALY. The cost impact analysis shows that the 1-week flucytosine course has an incremental cost of just over USD293 per patient per year compared to what is currently spent on standard of care. Sensitivity analyses suggest that the model is most sensitive to life expectancy and hospital costs, particularly infusion costs and length of stay.

**Conclusions:**

The addition of flucytosine as induction therapy for the treatment of cryptococcal meningitis in patients infected with HIV is cost-effective when it is used as a 1-week AmBd/5FC regimen. Savings could be achieved with early discharge of patients as well as a reduction in the price of flucytosine.

## Background

Cryptococcal infection is the most common cause of meningitis in adults living with HIV in sub-Saharan Africa. Cryptococcal meningitis (CM) is a serious opportunistic infection, which mainly affects persons with severe immunodeficiency. Most cases are observed in patients with CD4 T lymphocyte (CD4) cell counts < 100 cells/μL. In 2014, an estimated 280,000 people were reported to be positive for serum cryptococcal antigen globally and 220,000 new cases of cryptococcal meningitis occurred, of which sub-Saharan Africa contributed three-quarters of the cases and 135,000 deaths [1]. In South Africa, the number of cases of laboratory-diagnosed CM fell from 7140 in 2016 to 6636 in 2017 but the in-hospital case fatality ratio remained unchanged [2].

Generally, cryptococcal meningitis in HIV-infected patients in South Africa is treated initially with intravenous amphotericin B either alone or in combination with oral fluconazole [3]. Fluconazole and amphotericin B have been the mainstay of treatment for many years, however recent evidence has shown that the synergistic use of flucytosine improves efficacy and reduces toxicity [4]. The World Health Organization (WHO) has updated its guideline to recommend a combination induction phase of one week of intravenous amphotericin B and oral 5-flucytosine or, as an alternative, two weeks of oral flucytosine and fluconazole [5].

Lack of availability and access to 5-flucytosine is a common problem, especially in low-middle income countries. Also, the current international price renders it unaffordable in such countries [4, 6]. In South Africa, flucytosine is currently not registered by the South African Health Products Regulatory Authority (SAHPRA) and therefore is only available on a named-patient basis under Section 21 of the South African Medicines and Related Substances Act of 1965, as amended [7]. In anticipation of the registration of flucytosine, the Southern African HIV Clinicians Society recently released an updated guideline for the management of cryptococcal disease in PLHIV, which includes a recommendation for 1-week amphotericin B and flucytosine as induction therapy [8].

Findings from the Advancing Cryptococcal Meningitis Treatment for Africa (ACTA) trial [9] and a recent updated Cochrane Review [10] prompted an appraisal by the National Essential Medicines List Committee of the current treatment regimens used in South Africa including an updated cost-effectiveness and cost impact analysis [11]. The aim was to review the evidence and cost implications of introducing flucytosine as induction therapy for cryptococcal meningitis in HIV-infected adults in a public sector setting in South Africa. We present the key findings.

## Methods

A cost-effectiveness analysis from the perspective of the public payer, the South African government, was conducted using a decision tree analytic model measuring Life Years (LY) and Quality Adjusted Life Years (QALY) over a period of 25 years with a discount rate of 5%. Direct costs relating to treatment, hospital stay, pathology tests and managing adverse events were measured in South African Rands (ZAR) for 2018 and converted to USD. The model was developed based on survival estimates from the ACTA trial, a randomised controlled trial of flucytosine as induction therapy in HIV-infected adults with cryptococcal meningitis. The details of this study are available elsewhere [9]. Briefly, the study randomised HIV-infected adults with cryptococcal meningitis to one of five arms. The first was an entirely oral regimen of fluconazole 1200 mg daily with flucytosine 100 mg per kilogram daily for 2 weeks. This was compared to two regimens of one week of amphotericin B with two weeks of either flucytosine or fluconazole and two further regimens of 2 weeks of amphotericin B with two weeks of either flucytosine or fluconazole. After completing induction treatment, all the patients received fluconazole consolidation therapy (800 mg daily until initiation of antiretroviral therapy, then 400 mg daily until 10 weeks and 200 mg daily thereafter). Mortality rates were compared at 2, 4, and 10 weeks.

**Fig. 1:**
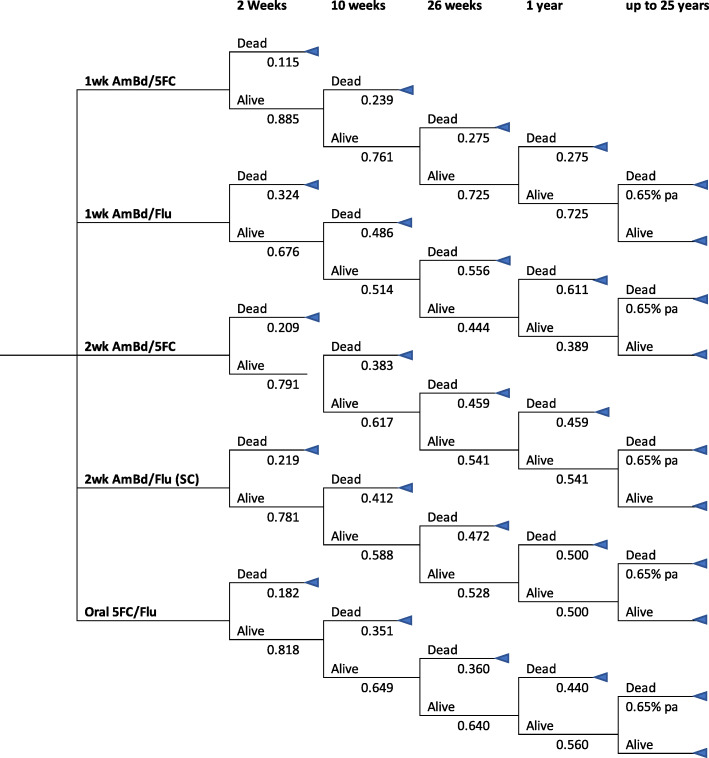
Schematic of decision tree with probabilities for each arm

A simple decision tree model was built in Microsoft Excel (2016), using survival probabilities from the main ACTA trial at time points of 2 and 10 weeks [9] and then 26 and 52 weeks from the 12-month follow up of a subset of the ACTA trial conducted in Malawi [12] with 2 health states: alive and dead. A schematic of the decision tree is presented in Fig. 1. For the first year the model used 1-week cycles to determine the probability of a patient being in either state as well as attribute costs and utilities. The total and incremental costs, life years and QALYs for 5 treatment options were calculated and compared to the lowest cost treatment arm, thereafter they were compared to the next less costly option. This was used to arrive at an incremental cost-effectiveness ratio (ICER) of cost/LYG (Life Years Gained) or cost/QALY. To assess the impact of uncertainty in the parameters used in the model, a deterministic univariate and two-way sensitivity analysis was conducted on key input parameters.

### Population and setting

The population of interest in this analysis were HIV-infected adults presenting with cryptococcal meningitis to a public health facility in South Africa. In general, these patients are treated at either a secondary (district or regional hospital) or tertiary and quaternary (specialist or teaching hospital) facility. Patients are usually treated in a general ward rather than an ICU or high-care setting.

### Study perspective

The study perspective was that of a provider, in this instance the South African Government, and therefore costs and outcomes were considered from the viewpoint of the public healthcare system. Direct costs such as medicines, hospital stay, consultations, adverse events, diagnostics and laboratory tests were considered.

### Interventions

The decision analytic model considered five treatment arms: (a) standard of care; 2-week course amphotericin B/fluconazole (2wk AmBd/Flu), (b) 2-week course amphotericin B/flucytosine (2wk AmBd/5FC), (c) short course; 1-week course amphotericin B/flucytosine (1wk AmBd/5FC) (d) oral course; 2-week oral fluconazole/flucytosine (oral) and e) 1-week course of amphotericin B/fluconazole (1wk AmBd/Flu).

### Time horizon

The time horizon of 25 years for this model was based on the average life expectancy of an HIV-infected patient at 35 years of age who is receiving ART from estimates from a recent South African collaborative study with a weighted average of CD4 counts and ratio of male: female demographics [13]. A sensitivity analysis was conducted to determine the impact of varying the time horizon.

### Discount rate

A discount rate of 5%, based on the South African Pharmacoeconomic Guidelines [14] was used for the outcomes and costs.

### Clinical and health outcomes

The model uses mortality rates from the full ACTA trial at at 2 and 10 weeks [9] and 6- and 12-month mortality rates from the Malawian long-term outcome ACTA study [12]. An average annual mortality rate for both men and women aged 35–39 years living with HIV in South Africa was determined from the Institute for Health Metrics and Evaluation (IHME) data tool based on the Global, regional, and national age-sex specific mortality tables for HIV/AIDS (1980–2017) of the Global Burden of Disease study (http://ghdx.healthdata.org/gbd-results-tool) [15].

Utilities were obtained from the Merry et al. cost-effectiveness study, whereby a utility of 0.5 was allocated to patients ill with CM during induction therapy (week 1–2), 0.8 to patients in the maintenance phase of CM (week 3–52) and 0.95 to patients well with HIV (year 2 onwards) [16]. A sensitivity analysis on these was conducted.

### Resource use and costs

All costs were determined for 2018. If necessary, prices or tariffs from previous years were adjusted by the average annual Consumer Price Index to bring them up to 2018 prices. All costs are presented in US dollars (USD) based on an average South African Rand to US dollar exchange rate for 2018 (Average R13.25) (Table 1) [22].

**Table 1:** Model input parameters

**Parameter**	**Value**	**Reference**
***Mortality rates***	**% (95% CI)**	
Mortality rates at 2 weeks		[9]
1-week AmBd/5FC	11.6 (5.7–17.5)	
1-week AmBd/Flu	32.4 (23.7–41.1)	
2-week AmBd/5FC	20.9 (13.4–28.3)	
2-week AmBd/Flu (SC)	21.9 (14.3–29.5)	
Oral 5FC/Flu	18.2 (13.2–23.3)	
Mortality rates at 10 weeks		[9]
1-week AmBd/5FC	23.9 (16.2–32.1)	
1-week AmBd/Flu	48.6 (39.4–57.9)	
2-week AmBd/5FC	38.3 (29.4–47.2)	
2-week AmBd/Flu (SC)	41.2 (32.3–50.4)	
Oral 5FC/Flu	35.1 (28.9–41.3)	
Mortality rates at 6 months		[12]
1-week AmBd/5FC	27.5 (16.3–44.1)	
1-week AmBd/Flu	55.6 (40.4–72.0)	
2-week AmBd/5FC	45.9 (31.6–63.1)	
2-week AmBd/Flu (SC)	47.2 (32.6–64.5)	
Oral 5FC/Flu	36.0 (26.3–47.9)	
Mortality rates at 12 months		[12]
1-week AmBd/5FC	27.5 (16.3–44.1)	
1-week AmBd/Flu	61.1 (45.8–76.7)	
2-week AmBd/5FC	45.9 (31.6–63.1)	
2-week AmBd/Flu (SC)	50.0 (35.1–67.1)	
Oral 5FC/Flu	44.0 (33.7–55.9)	
**Parameter**	**Basecase (range)**	
Mortality rate per annum (year 2 onwards)	0.65% (0.57–0.72)	[15]
***Utilities***		
Well with HIV	0.95 (0.8–0.98)	[16] (a)
Ill with CM (induction)	0.5 (0.43–0.58)	[16] (a)
Well with CM (maintenance)	0.8 (0.68–0.92)	[16] (a)
***Length of stay (LOS) (days)***	17 (7–10)	[9] (b)
***Patient weight (kg)***	60 (45–75)	(c)
***Medicine costs (per dose)***	**USD**	
Amphotericin B Deoxycholate (50 mg)	0.63	[17]
Fluconazole (200 mg)	0.13	[17]
Flucytosine (500 mg)	34.02	(d)
Saline 0.9% 1 l	0.62	[17]
Potassium chloride inj (15% in 10 ml)	0.13	[17]
Potassium tablets 600 mg	0.08	[17]
Magnesium tablets 250 mg	0.02	[17]
Flucloxacillin (50 mg)	0.04	[17]
Ceftriaxone 1 g vial	0.44	[17]
Ampicillin 500 mg vial	12.14	[17]
Ciprofloxacin 400 mg vial	2.76	[17]
***ART first line total cost per year***	251	[18]
***Infusion fee per day***	15.25 (0–113.6)	[19] (e)
***Hospital cost per day***	85.06 (73.41–189.39)	[19] (f)
***Laboratory monitoring***		
Serum potassium	2.17	[20]
Serum creatinine	2.17	[20]
Serum magnesium	2.17	[20]
Haemoglobin	4.38	[20]
Full blood count	4.14	[20]
Blood draw	2.79	[20]
***Lumbar puncture***		
Diagnostic	60.75	[20, 19]
Therapeutic	46.02	[20, 19]
Blood transfusion	170	[21]
**Discount rate**	5% (0–10%)	[14]

(a) Assumption of ±15% of basecase

(b) Assumption of 1 week and 10 days LOS

(c) Personal communication from Adult Level Essential Medicines List Committee

(d) Personal communication on buy-out price in Western Cape

(e) From no cost for infusion fee to cost in private sector facility

(f) Cost of Level 2 public sector facility to cost in private sector general ward facility

Medicine prices were sourced from the public sector Master Procurement Catalogue for September 2018 [17]. The price of flucytosine was obtained from a quote for named-patient use in Western Cape, South Africa (National Department of Health communication on file, T Leong 2019)**.** Pre-emptive hydration and electrolyte supplementation were included as recommended in the WHO guidelines [5] and as per the ACTA trial [9]. Laboratory costs were obtained from the NHLS State Price List 2017 [20] and lumbar puncture (LP) costs were determined either as a diagnostic cost (including rapid antigen assay and culture) or a therapeutic cost (to relieve raised intracranial pressure). The lumbar puncture costs included a procedure fee, consultation, and laboratory tests. The utilisation of laboratory tests for monitoring was based on the WHO guidelines of twice weekly monitoring of potassium, magnesium and creatinine, weekly haemoglobin monitoring and full blood count for flucytosine monitoring (twice weekly for duration of treatment).

Base-line hospital costs were determined for a Level 2 facility with an in-patient stay of 17 days as per the ACTA trial [19]. This took into consideration additional length of stay in a proportion of patients due to adverse drug reactions (ADRs) or failure to respond to treatment. ADR costs incorporated the costs of managing anaemia and neutropenia. Treatment of anaemia included the cost of whole blood, administration sets and a delivery fee [21]. The utilisation rate of antibiotics to treat neutropenia was taken from the Chen et al. study although only antibiotics that are available in South Africa on the EML were included [23].

Annual antiretroviral therapy (ART) treatment costs for 2018 were obtained from a recent systematic review of per patient costs for HIV care in South Africa [18]. The model was not designed as a markov model and therefore did not determine the progression of patients from first to second line treatment. It was assumed that all patients stayed on first line ART throughout the duration of the model.

### Cost impact analysis

The cost impact analysis was based on 7,497,774 HIV-infected patients in 2018 [24] with a CM prevalence of 93/100,000 population based on confirmed cases of CM from the GERMS 2017 report [2]. Past trends seem to suggest that cases of CM may be decreasing year on year. It was assumed that not every patient would access flucytosine in 2018 and therefore an uptake of 60% was selected for the base case with the balance of patients remaining on standard of care. The results of the analysis were based on total costs per patient in the first year taking into consideration the probability of patients dying, and thus not requiring further treatment.

### Assumptions

For managing relapses or recurrences, the WHO Guidelines recommend restarting the induction phase as per the initial recommendations. This model did not specifically include relapses or recurrences; however, these were included in the total count of cases of CM per year and assumed to have similar costs to first infections. Although paradoxical cryptococcal immune reconstitution inflammatory syndrome does occur with an estimated frequency of 10–50% [25] in patients initiating ART in all the treatment regimens and this condition is associated with a high mortality, it is assumed that this is already included in the overall mortality rate attributed to CM as per the outcomes based on the clinical trial data.

It was assumed that patients had one diagnostic LP and one therapeutic LP regardless of which regimen they were treated with. In the ACTA trial patients received on average 3 LPs, at baseline and on days 7 and 14, however this was conducted under clinical trial conditions and the WHO guidelines do not recommend routine follow up LPs in resource limited countries [4] A sensitivity analysis was conducted to assess the impact of changing the number of LPs.

Generally, there is no vial sharing for amphotericin B for patients, so it was assumed that 2 vials per dose were used. This was based on expert opinion of an estimated patient weight of 60 kg at a dose of 1 mg/kg/day (personal communication, Adult Hospital Level Essential Medicines List Committee meeting May 2019). Although the average weight of a patient is usually assumed to be 70 kg, in the ACTA trial the average weight range was 50-53 kg across treatment cohorts. Differences in weight and the opportunity for vial sharing were tested in the sensitivity analysis.

For the base-case it was assumed that all patients stayed in hospital for an average length of stay of 17 days (range 12.27–19.31 across all arms) based on the utilisation data from the ACTA trial [23].

There is little published information on the use of antibiotics as treatment of neutropenia in patients with CM so assumptions were based on availability of these medicines on the Essential Medicines List. It was assumed that all patients who experienced neutropenia requiring IV antibiotics were still in hospital for CM treatment and therefore did not incur additional hospital costs, only those of the antibiotic treatment.

## Results

### Base-case analysis

**Fig. 2:**
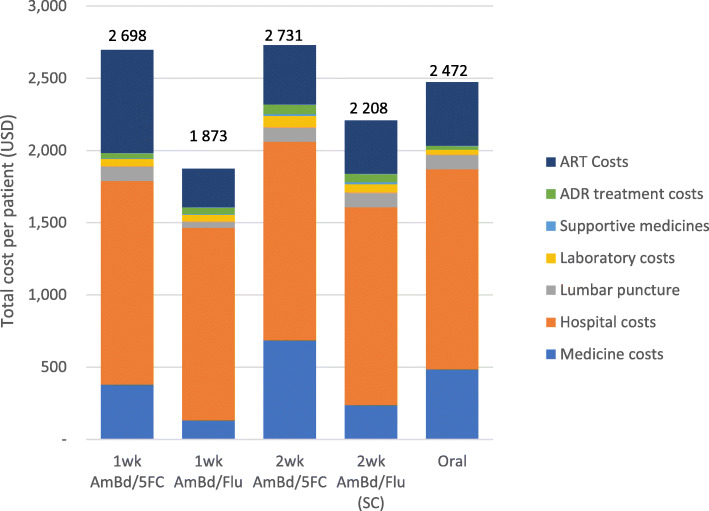
Total cost per patient per treatment arm

When the proportion of patients alive at each time point in each arm is taken into consideration, the highest total treatment costs were in the 2-week AmBd/5FC arm, followed by the 1-week AmBd/5FC, then the 2-week oral regimen and standard of care with the lowest total cost in the 1-week AmBd/Flu arm. Monitoring costs are highest in the 2-week Am/5FC arm followed by the standard of care. Supportive medicine costs are lowest in 1-week courses with no additional costs for the oral regimen. The 1-week AmBd/5FC arm has the highest ART costs due to more people being alive throughout the model (Fig. 2).

The total LYs and QALYs for each treatment arm are shown in Table 2. As expected, the 1-week flucytosine arm has the highest numbers of LYs and QALYs, followed by the oral regimen, the 2-week flucytosine arm and 2-week standard of care with the lowest in the 1-week amphotericin B/fluconazole arm.

**Table 2:** Total LYs, QALYs and Costs per treatment arm

Treatment arm	LY	QALY	Cost (USD)
1-week AmBd/5Flu	8.67	8.15	1873
2-week AmBd/Flu (SC)	11.10	10.44	2208
Oral 5FC/Flu	12.42	11.69	2472
1-week AmBd/5FC	16.01	15.08	2698
2-week AmBd/5FC	11.99	11.28	2731

When considering the costs and outcomes of each treatment arm, it is apparent that the lowest cost treatment (1wk AmBd/Flu) also has the poorest outcomes. If the treatment arms are compared to the lowest cost, lowest QALY regimen then the 1-week AmBd/5FC has the lowest ICER at USD119/QALY, followed by the standard of care (Table 3). The oral regimen has the highest ICER at USD169/QALY. The 2-week AmBd/5FC is absolutely dominated by the 1-week AmBd/5FC arm in that it has a higher cost and lower QALYs. The 1-week AmBd/5FC has extended dominance over the standard of care and oral regimen with a lower ICER compared to the lowest cost regimen, despite having higher costs. Therefore, the only ICER that should be considered is that of the 1-week AmBd/5FC compared to the lowest cost regimen of 1-week fluconazole.

**Table 3:** QALYs, Costs and ICERs per treatment arm

Treatment arm	Cost (USD)	QALY	ICER
1-week AmBd/Flu	1873	8.15	–
2-week AmBd/Flu (SC)	2208	10.44	Weakly dominated^a^
Oral 5FC/Flu	2472	11.69	Weakly dominated^a^
1-week AmBd/5FC	2698	15.08	119
2-week AmBd/5FC	2731	11.28	Dominated^b^

^a^ Weakly dominated; where mutually exclusive programmes are compared, and one option has a lower ICER than the next less costly option

^b^ Dominated; where at least one other treatment option has lower costs and higher benefits

### Sensitivity analysis

**Fig. 3:**
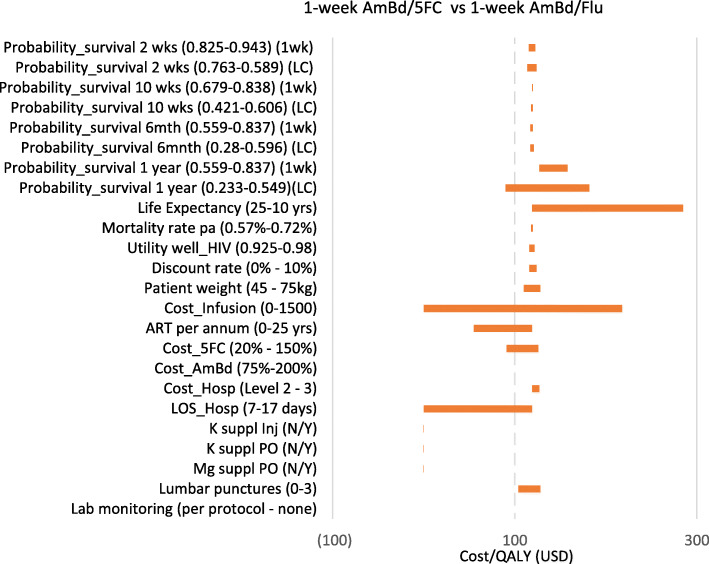
Tornado diagram of ICER sensitivity for 1wk AmBd/5FC compared to 1wk AmBd/Flu

When a sensitivity analysis was conducted to assess the uncertainty in the model it was found that varying most parameters did not have a substantial impact on the outcomes and the ICERs mostly remained below USD300/QALY. The sensitivity analysis focused mainly on the 1wk AmBd/5FC regimen compared to the 1wk AmBd/Flu as this reflected the lowest ICER in the analysis. The model is most sensitive to cost of infusions fees, hospital length of stay, life expectancy and probability of survival at 12 months in the lowest cost arm. The model was less sensitive to the inclusion of annual ARV treatment costs and then the cost of flucytosine (Fig. 3). A two-way sensitivity analysis of the key parameters that showed uncertainty in the one-way sensitivity analysis was conducted using a combination of infusion fee cost, length of stay, life expectancy, inclusion of ARV treatment costs and the price of flucytosine. The model was most sensitivity to the probability of survival at 12 months in the 1-week fluconazole arm and life expectancy where the ICER for 1-week 5FC compared to 1-week fluconazole increased to USD12,225/QALY at a probability of 0.233 (lower 95% CI) and life expectancy of 10 years. The lowest ICER achieved (USD27/QALY) was when the ARV treatment costs were excluded and the price of flucytosine was reduced by 80%. If the model was run for only the first 12 months the ICER increases to USD2072/QALY.

The inclusion of costs for treatment of anaemia (blood transfusions) or neutropenia (antibiotics) does not impact substantially on the ICER outcomes, nor the inclusion or exclusion of use of potassium or magnesium supplementation. Increasing the number of LPs had very little impact on the ICERs. Increasing patient weight resulted in higher costs but only slightly increased the ICER. When the annual ART costs are removed, the 1wk AmBd/5FC still remains the most cost-effective although the ICER is reduced to USD55/QALY.

The model is sensitive to the price of flucytosine with the costs of the 1wk AmBd/5FC and oral arm nearing those compared to standard of care at a 50% price discount, although the extended dominance over the oral regimen and standard of care remains.

When the cost of infusion fees is increased beyond USD 37, both the 1-week flucytosine and oral course are dominant over the standard of care although not at the standard cost of tertiary level facility infusion fees (USD17 per infusion). Although other published cost-effectiveness analyses included an infusion fee as a separate cost item, in South Africa it most likely included in the hospital daily rate. The impact of excluding the infusion fee cost was assessed and found to reduce the ICER but not to less than USD104/QALY. Varying the discount rate from 0 to 10% also had little impact on the ICER.

Due to the trial protocol in ACTA the average length of stay (LOS) in the trial was 17 days regardless of which treatment arm patients were in. It is possible that patients who are well enough to be discharged may leave sooner if they are on the 1-week AmBd/5FC or oral regimen. This has an impact on the model outcomes so that if the length of stay in hospital is reduced to 10 or 7 days for either the 1-week or oral flucytosine course, the ICERs are further reduced with a lower cost and higher QALYs compared to the 2-week courses of treatment.

### Cost impact analysis

The incremental costs for all the related costs (including hospital stay, monitoring, ADRs) for the first year of treatment were presented as well as the impact if only medicine costs are considered. Assuming the base case inputs, modelling the same LOS (17 days) for all treatment arms, and if up to 60% patients were to switch to that particular regimen in the first year, the greatest total cost impact for 2018 is with the 2-week AmBd/5FC course of treatment (USD11 411,071), followed by the 1-week course (USD11 274,242), the oral regimen (USD10 332,485), the current standard of care (USD9 228,883) with the 1 week fluconazole course having the lowest cost impact at USD7 825,941. Although the 1-week AmBd/Flu course has the lowest cost, it was decided not to include it in the cost impact analysis due to the higher mortality and the fact that the 2-week AmBd/Flu course is the current standard of care.

If 60% of patients were switched to the flucytosine regimens, the average additional cost per patient (total costs) over what is currently spent on standard of care would be USD293 per patient per year for the 1-week flucytosine course (Table 4). The incremental cost for medicines only would be USD596651. As the price of flucytosine is reduced so the incremental cost decreases. At a 25% price reduction the average incremental cost per patient is USD257 and becomes USD221 at a 50% price reduction. The incremental cost of medicines only becomes cost neutral at a flucytosine pack price of around USD113 (USD94 per week of treatment). If the estimated uptake of flucytosine is increased to 70%, the cost impact increases to USD2 386,252 in total (USD341 per patient). When the uptake of flucytosine is reduced to 50% the incremental cost is USD1 704,466 (USD244 per patient).

**Table 4:** Incremental cost impact (total and per patient) compared to lowest cost and standard of care

Total Inc. impact (2018)	Total Incrementalcost (USD)	Incremental costper pt (USD) Yr 1
**1wk AmBd/5FC**		
vs 1wk AmBd/Flu (LC)	9,600,890	1373
vs 2wk AmBd/Flu (SC)	2,045,359	293

In addition, the model is sensitive to whether an infusion fee is included or not with the incremental cost increasing slightly for the 1-week course when an infusion fee is not included. As the infusion fee increases so the cost of the 1-week flucytosine course becomes cost neutral. The greatest impact is seen when the LOS is reduced for the 1-week AmBd/5FC regimen assuming that patients may be discharged sooner. If the LOS is reduced to 10 days or 7 days, the 1-week courses become increasingly cost saving.

## Discussion

The introduction of flucytosine as the mainstay of induction therapy for the treatment of cryptococcal meningitis has been hampered by issues of affordability and sustainable access, particularly in LMICs in Africa [26]. This study, using outcomes data from the ACTA trial, has shown that it is most cost-effective to treat patients with a 1-week short course of flucytosine and amphotericin B. In addition to improved mortality outcomes, this treatment option provides an opportunity for patients to be discharged earlier than 2 weeks, resulting in a potential reduction in costs, as well as a reduction in toxicity related to shorter duration of amphotericin B treatment.

Whilst the 1-week AmBd/Flu (currently lowest cost per patient) would also incur lower costs due to a shorter hospital stay, it has the highest mortality rate of all the treatment arms. Theoretically this presents a possible decision that could be made to choose this regimen over the other arms despite the mortality loss in favour of a significant savings, although it is unlikely the decision-making committee would recommend such an option.

The model was sensitive to reductions in length of stay for the short course flucytosine treatments. Data on length of stay in patients on flucytosine from a study in the Eastern Cape, South Africa, indicates that the average LOS post introduction of flucytosine was reduced to a median of 14 days (IQR 14–27) from 20 days (IQR 9–21) in the pre-flucytosine cohort, however this was not significant [27]. The fluconazole dosing in the ACTA trial differs from that of the WHO Guidelines [5]. The initial oral regimen used in the trial is 1200 mg fluconazole for 14 days (or 7 days in the case of the short course). The WHO Guidelines recommend 800 mg per day. However, using the WHO regimen in the sensitivity analysis did not impact the outcomes of the model.

Amphotericin has widely been implicated in the development of nephrotoxicity and anaemia [28, 29]. The development of nephrotoxicity or anaemia is associated with increased 10-week mortality and therefore these are important ADRs to monitor and manage [30]. The probability of patients developing Grade IV anaemia was based on the ACTA trial and it was assumed that these patients all received a transfusion. The utilisation of blood units was obtained from the Chen et al. study [23]. However, in a study conducted in South Africa, it was noted that of all the patients with Grade IV anaemia, only around 20% of those actually received a transfusion [28]. Nephrotoxicity is largely managed by pre-emptive saline, hydration and electrolyte replacement. However, if patients do develop nephrotoxicity Grade III or IV, management begins with omitting the next AmBd dose and giving additional fluids. If creatinine levels are still rising, then the patient is either moved to alternate day dosing or treatment is stopped altogether [29, 31]. In neither the Bicanic nor ACTA trials were any mention made of patients who required dialysis as management of nephrotoxicity. Similarly, renal dialysis was considered to be very rare in the local setting (personal communication, Adult Essential Medicines List Committee meeting May 2019). It is possible an extended length of stay may be required to complete the course of treatment.

A limitation of our model was the assumption that there would be no additional changes in health or costs in the following years. However, relapses do occur, particularly in patients who default on their fluconazole maintenance therapy or who stop their ARVs [31, 32]. Therefore, it is possible that some patients will incur additional expenses in subsequent years. However, we assumed this would be similar in incidence across all arms of the model and presumably with similar costs. Another limitation is the simplicity of the model where additional analyses such a probabilistic sensitivity analysis and markov modelling could more accurately reflect the changes in treatment and health outcomes over the duration of the model.

Cost-effectiveness thresholds vary by country and in many cases are not explicit. The commonly used WHO threshold of one to three times per capita GDP has been increasingly refuted [33] and more recent estimates have suggested a threshold of between USD1175/QALY and USD4714/QALY for South Africa [34]. In 2016, South Africa conducted an investment case analysis to determine the optimal mix of HIV interventions required to reach the 90–90-90 targets. The baseline was the HIV programme at the time under the committed budget for 2016–2018. The ICERs for 16 different interventions were determined and then ranked by increasing technical efficiency to assess which were likely to achieve the greatest benefit within the budget [35]. Through a revealed willingness to pay for the optimal mix of interventions, a cost-effectiveness threshold for HIV treatment in South Africa was determined to be USD547–872 per life-year-saved [36]. Although there is no explicit threshold in South Africa, the ICERs calculated in this study would be considered cost-effective as they are below the lower limit of the thresholds described above. Economic evaluations in both the USA [16] and Africa [23, 37, 38] have shown that flucytosine is cost-effective compared to current standard of care, although affordability through budget impact analysis had not been considered. In this study we estimated the impact on direct medical costs and determined a price at which the introduction of flucytosine would become cost-neutral to introduce to the essential medicines list in South Africa. Finding a cost-neutral price as a benchmark was requested by the decision-makers to inform price negotiations with the manufacturer when bringing flucytosine to the market. Although it is not always possible to properly compare pricing across countries and exchange rate changes must be considered, the price of flucytosine in South Africa was found to be higher than in other U/LMIC countries (India, Poland, Uganda) so efforts were required to try to align with these and other countries.

The outcomes and resource utilisation in a general clinical practice setting such as that in the public sector in South Africa may differ from other countries and it is recommended that if flucytosine is included on the Essential Medicines List in South Africa, a monitoring and evaluation study is conducted to verify these outcomes and costs.

## Conclusions

This updated cost-effectiveness analysis in a public health setting in South Africa confirms that the addition of flucytosine as induction therapy in the treatment of cryptococcal meningitis in patients infected with HIV is cost-effective when used as a 1-week regimen with an ICER of USD119/QALY, compared to the lowest cost fluconazole regimen. The model is most sensitive to changes in costs rather than outcomes with the greatest impact seen where cost reductions can be achieved by reducing the hospital length of stay and infusion fee costs.

Although the incremental cost of flucytosine compared to current standard of care is in the region of USD600 000 per annum, savings could be achieved with early discharge of patients as well as a reduction in the price of flucytosine.

Improved access to flucytosine at a reasonable cost in South Africa, and particularly with the introduction of the 1-week course, has the potential to reduce the length of stay from 14 days to 7 days of treatment with an associated reduction in mortality. With the availability of robust evidence and cost-effectiveness data, access to flucytosine in LMICs and UMICs as an essential medicine is imperative and affordability can be achieved with appropriate price negotiations.

## Data Availability

The datasets analysed during the current study are available in the following repositories: Master Procurement Catalogue (http://www.health.gov.za/index.php/component/phocadownload/category/196) and the Institute for Health Metrics and Evaluation (IHME) data tool (http://ghdx.healthdata.org/gbd-results-tool). Any additional datasets generated for use and/or analysed during the current study are available from the corresponding author on reasonable request.

## Abbreviations

5FC: 5-Flucytosine
ACTA: Advancing Cryptococcal Meningitis Treatment for Africa
ADRs: Adverse drug reactions
AmBd: Amphotericin B
ART: Antiretroviral therapy
ARV: Antiretrovirals
BIA: Budget impact analysis
CD4: CD4 T lymphocyte
CM: Cryptococcal meningitis
Flu: Fluconazole
ICER: Incremental cost effectiveness ratio
IHME: Institute for Health Metrics and Evaluation
LOS: Length of stay
LP: Lumbar puncture
LYs: Life years
LYG: Life years gained
QALY: Quality adjusted life year
SAHPRA: South African Health Products Regulatory Authority
SANBS: South African National Blood Service
SC: Standard of care
UPFS: Uniform Patient Fee Schedule
WHO: World Health Organisation
